# Supplementary material for: Transcriptional reprogramming of natural killer cells by vaccinia virus shows both distinct and conserved features with mCMV
Source: Front Immunol. 2023 Feb 23;14:1093381. doi: 10.3389/fimmu.2023.1093381 (PMC9995584; doi:10.3389/fimmu.2023.1093381)
Supplement: Supplementary file 1 [file DataSheet_1.docx]

Supplementary Material


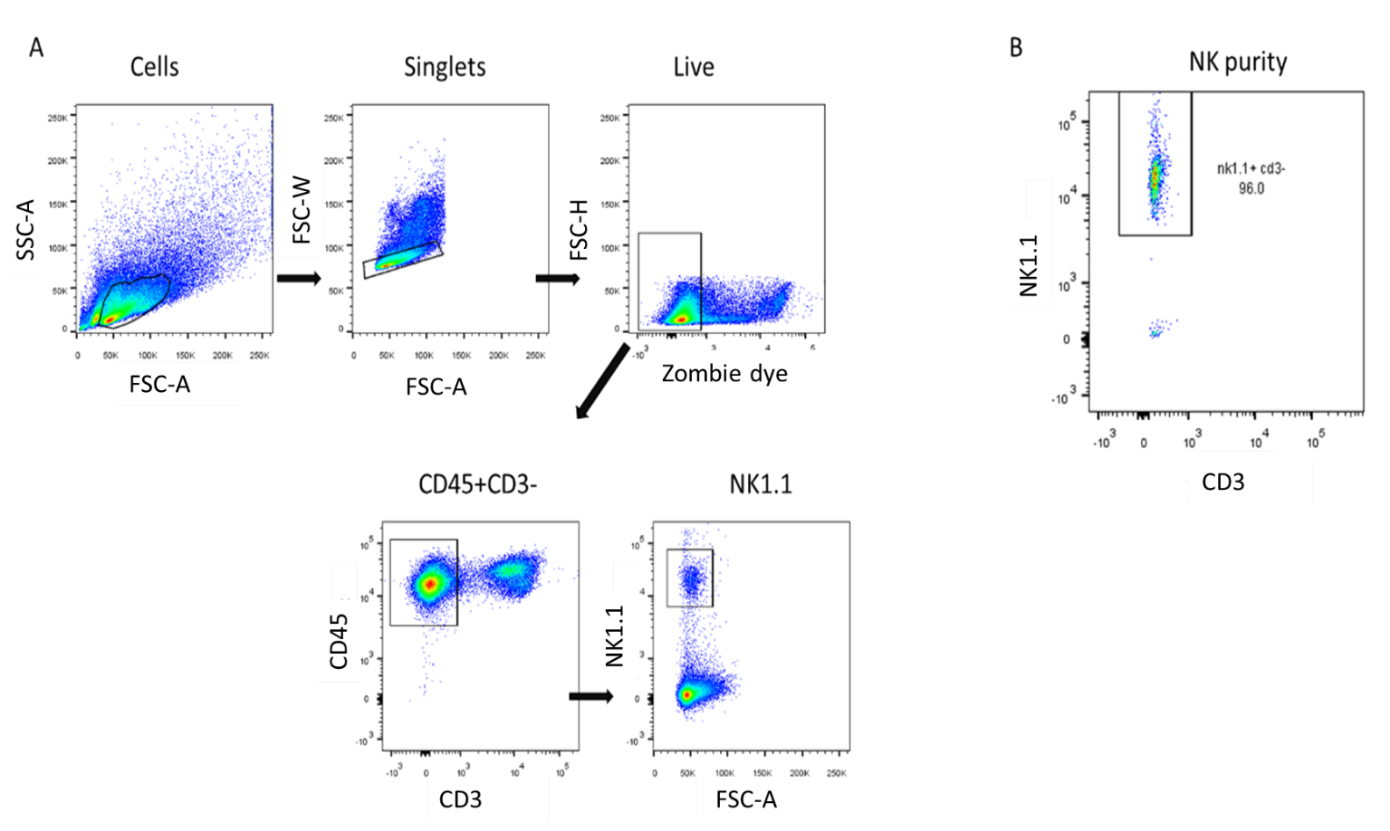


**Supplementary Figure 1**. **Gating strategy for NK cell isolation by FACS and purity check**. (A) Debris were gated out and viable single cells that were negative for CD3, and positive for CD45 and NK1.1, were isolated by FACS in sterile PBS. (B) The purity of the NK cells was checked by running a sample of the sorted cells on the flow cytometer.


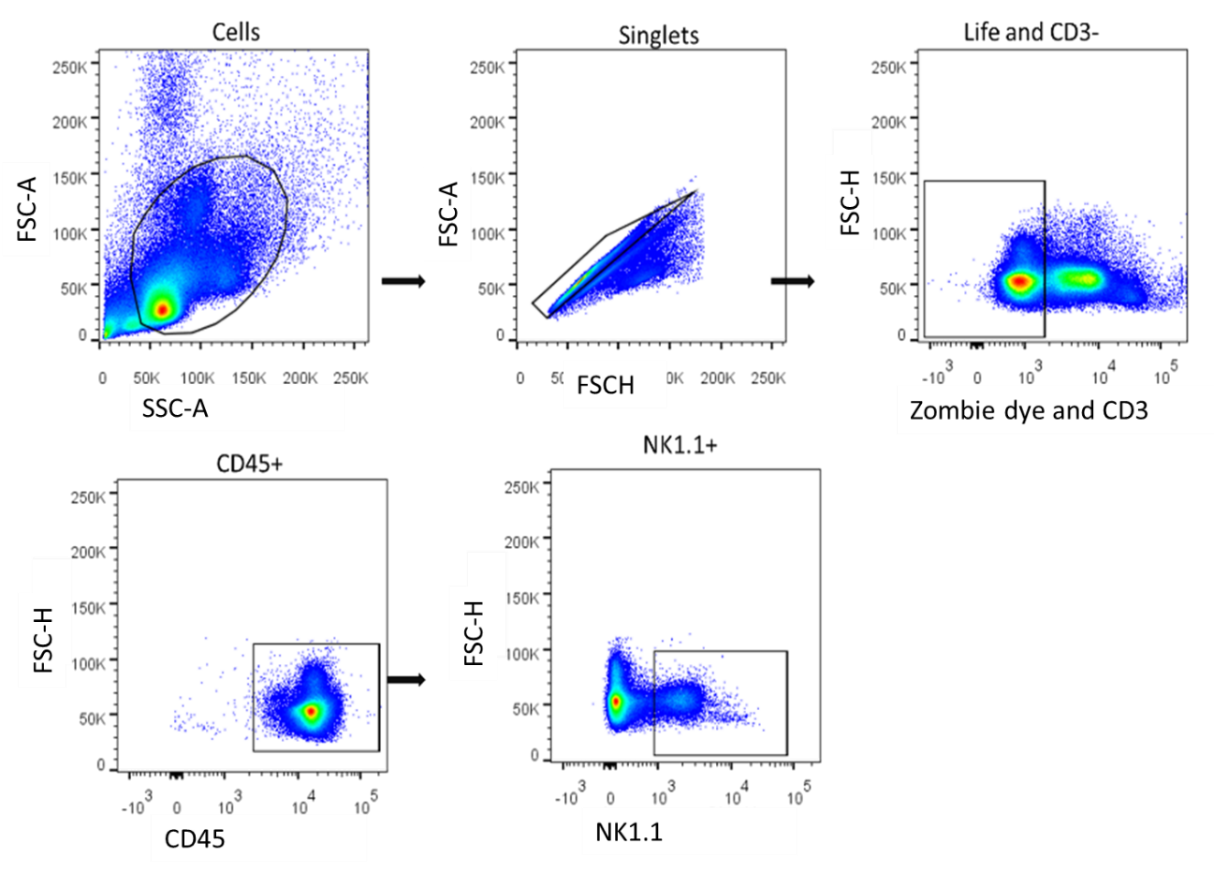


**Supplementary Figure 2.** Common gating strategy used for NK phenotyping by FACS. Debris were gated out and then viable single cells negative for CD3, and positive for CD45 and NK1.1 were gated.


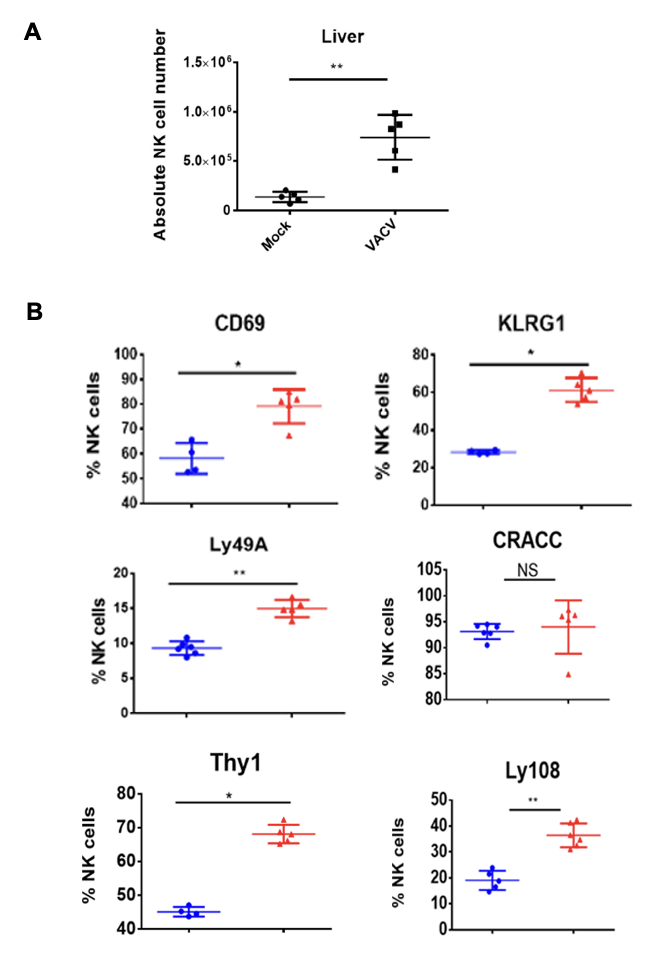


**Supplementary Figure 3.** C57BL/6 mice were mock-treated or infected i.n. with VACV and 6.5 d later (A) hepatic NK cells were counted, B) Protein expression on hepatic NK cells assessed by FACS from mock (blue) and VACV-infected mice (red). The percentage of NK cells expressing the indicated surface protein is shown. Error bars represent ± SD, statistical significance was assessed with a Mann-Whitney test (*p<0.05, ** p<0.01, ***p<0.001).

**Tables**

| Antigen | Fluorochrome | Clone | Company cat. number |
| --- | --- | --- | --- |
| Fixable Viability Dye | Zombie violet |  | Biolegend #423113 |
| CD3 | BV421 | 145-2C11 | Biolegend #100341 |
| CD45 | BV650 | 30-F11 | Biolegend #103151 |
| NK1.1 | APC-Cy7 | PK136 | Biolegend # 108724 |
| CD16/CD32 |  | 2.4G2 | BD #553142 |
| CD27 | APC | LG.3A10 | Biolegend # 124211 |
| CD11b | FITC | M1/70 | Biolegend #101205 |
| KLRG1 | BV510 | 2F1 | BD #740156 |
| Thy-1.2 | PerCP | 30-H12 | Biolegend #105321 |
| CD69 | PE | H1.2F3 | Biolegend #104507 |
| Ly49C/I | FITC | 5E6 | BD # 553276 |
| Ly49H | AF647 | 3D10 | BD #62207 |
| CD107a | PE | 1D4B | Biolegend # 121611 |
| Ly49A | FITC | YE1/48.10.6 | Biolegend 116805 |
| Ly49F | APC | HBF-719 | Miltenyi Biotech 130-104-293 |
| CD319 | PE | 4G2 | Biolegend#152005 |
| Ly49D | FITC | 4E5 | BD #555313 |
| Ly49G2 | APC | 4D11 | BD #555316 |
| LY108 | PE | 330-AJ | Biolegend #134605 |
| CD49b | FITC | HMα2 | Biolegend 103503 |
| gp49B | PE | H1.1 | Biolegend 144904 |
| CXCR6 | APC | SA051D1 | Biolegend #151104 |

**Table 1****.** Antibodies used for Flow cytometry

**
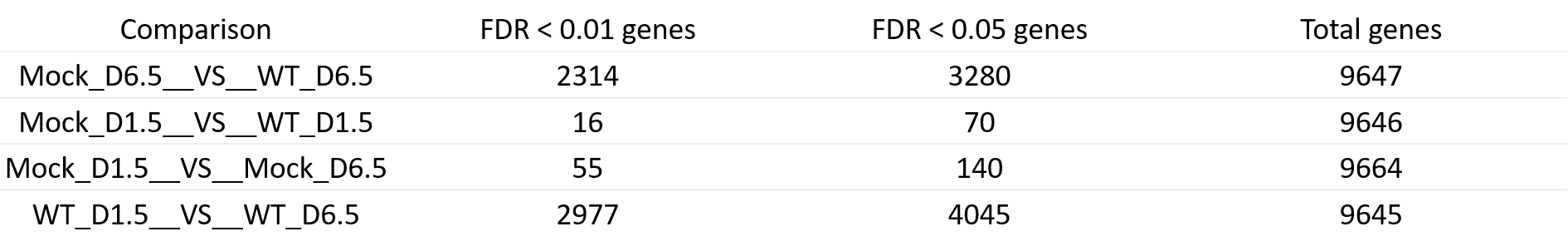
**

**Table 2.** Number of genes differentially expressed for the indicated pairwise comparisons during RNA-seq study of murine NK cells. Groups of B6 mice (n=4) were i.n. infected with WT VACV or vehicle control for 1.5 or 6.5 d.p.i.. Differential expression of transcripts was analysed following 4 pairwise comparisons, as indicated in the first column. The number of transcripts differentially expressed in a statistically significant manner is indicated in the second column (FDR<0.01) and in the third column (FDR<0.0.5). The total number of genes detected is indicated in the last column**.**
